# Decoding Mohs Micrographic Surgery Through an Artificial Intelligence-Powered Search Analytics Approach

**DOI:** 10.7759/cureus.90093

**Published:** 2025-08-14

**Authors:** Landon E Ebbert, Joshua L Ebbert, Justin L Knapp, Madelin R Morris, Alejandro M Holle, Maggie R Donovan, Lisa D Grunebaum

**Affiliations:** 1 Otolaryngology, Mayo Clinic Alix School of Medicine, Phoenix, USA; 2 Physics and Astronomy, Brigham Young University, Provo, USA; 3 Biology, Brigham Young University, Provo, USA; 4 Otolaryngology - Head and Neck Surgery, Mayo Clinic, Phoenix, USA

**Keywords:** artificial intelligence, cutaneous skin cancer, internet, mohs micrographic surgery, search analytics

## Abstract

Background

Patients often seek information online regarding Mohs micrographic surgery. Google’s "People Also Ask" algorithm provides insights into patients’ questions, compiling frequent queries through machine learning. The experimenters aimed to utilize modern search analytics methods with the integration of machine learning qualitative data analysis to determine patient concerns and questions surrounding Mohs surgery.

Methodology

Rothwell’s classification system and the Journal of the American Medical Association benchmark criteria were used to evaluate question-website pairs generated by Google’s “People Also Ask” function. Statistical analyses assessed machine learning classification performance and relationships between search terms, question types, and website categories.

Results

The highest proportion of questions belonged to technical details (19.8%), follow-up care (12.5%), and indications (11.6%). Among website types, government sites had the highest Journal of the American Medical Association benchmark scores. Compared with human reviewers, machine learning algorithms exhibited kappa values as high as 0.86.

Conclusions

Mohs surgery patients are interested in understanding the technical details of their condition and procedure. Healthcare professionals should address these concerns to ensure greater compliance with treatment protocol and mitigate the risk of iatrogenic trauma. In qualitative research applications involving a panel of reviewers, high-performing large language models offer a suitable component of a review board and complement human expertise.

## Introduction

Among the most common dermatological procedures performed is Mohs micrographic surgery (MMS). In 2013, it was reported that Medicare-funded MMS procedures amounted to 700,262 cases, and with the increasing incidence of cutaneous cancers, this number has likely only grown [1-2]. The high volume of MMS procedures is also due to its high success rate of up to 99% and conservation of tissue through microscopic precision [3].

MMS is often indicated for the surgical excision of recurrent, high-risk, large dermatological cancers such as basal cell carcinoma, cutaneous squamous cell carcinoma, and melanoma in situ when other therapies - standard excision, cryotherapy, topical therapies - have not been effective or advised [4]. Patients often seek information online regarding MMS, reflecting broader trends in health-related internet use, with 58.5% of adults searching for medical information in 2022 [5]. To optimally deliver care, it is imperative that healthcare professionals are aware of such questions. Patients’ questions can help inform the healthcare team of the goals of care, obstacles that must be overcome for optimal health outcomes, and concerns that should be appropriately addressed. Google’s "People Also Ask" (PAA) algorithm provides insights into common patient questions, compiling frequently searched queries through machine learning (ML) tools such as RankBrain and BERT [6].

Previous studies have successfully used PAA data to analyze patients’ questions and evaluate the quality of online materials [7]. This study primarily aims to use modern internet search analytics and Google PPA data to characterize patient concerns regarding MMS and online MMS resources encountered pre- and postoperatively. Existing literature is characterized by investigations into the quality and readability of dermatologic and specifically MMS education modalities, yet none have used PAA data to fully investigate both pre- and post-surgery materials for MMS patients [8-12]. Additionally, this study further adds to the literature to better understand the perspectives, needs, and experiences of prospective and actual MMS and Mohs reconstruction patients [5,13-14].

A secondary aim of this study is to evaluate ML and LLM-based methods that can enhance the accuracy and scalability of qualitative data classification in future internet search analyses, reducing reliance on manual human review. To determine the types of questions that patients ask regarding MMS, a team of reviewers must read and classify hundreds of questions. Such a time-intensive process inevitably results in errors from volume-induced data collection fatigue. Additionally, human reviewers may be inconsistent in the application of rules designed to aid in making classifications, and this further results in judgment-based discrepancies.

Recent advancements in artificial intelligence, particularly ML and its subsidiary, deep learning (DL), offer sundry tools for automating and accelerating qualitative data analysis. ML leverages sophisticated algorithms and statistical models to analyze and draw inferences from patterns in data. DL constitutes the use of layered artificial neural networks for extracting high-level features from large and complex collections of data. Large language models (LLMs), which are powered by DL, are designed to process and generate human language using vast amounts of text data, making them effective for tasks such as literature reviews and summarizing large bodies of research.

The growing availability of cloud resources has made it feasible to apply these techniques across a wide range of fields. Hasan et al. proposed a framework for incorporating LLMs into systematic reviews, highlighting their potential to streamline qualitative data analysis [15]. In this study, we extend the proposed protocol by applying it to patient-facing qualitative data and evaluating its performance against well-established ML algorithms, including K-nearest neighbor (KNN), logistic regression (LR), and decision tree classifier (DTC). We investigate how these ML algorithms, including LLMs, perform in internet search analytics, recognizing the broader applicability of this framework across diverse research fields, both medical and non-medical.

## Materials and methods

PAA questions and website extraction

This study methodology was adapted from Suresh et al.’s study, in which PAA questions were categorized and corresponding websites were analyzed for quality [7]. Six search terms ("Mohs surgery", "Mohs reconstruction", "Mohs surgery repair", "Mohs procedure", "skin cancer surgery", "skin cancer removal") were entered into the Google engine in February 2024. The chosen search terms were used to account for differences in health literacy among patients searching the web. Google’s PAA function generated 85 unique questions per query, extracted using the SEO Minion extension (Axeman Technology Solutions, Version 3.7, Mumbai, India). This study was deemed exempt by the Mayo Clinic IRB.

Question and website classification

Each extracted question was categorized into one of three categories - Fact, Policy, or Value -according to Rothwell’s system [16] before subcategorization into one of 13 topics relating to MMS (Table 1) [5]. Linked websites were categorized by source type (e.g., Commercial, Academic, Medical Practice). Two reviewers independently classified extracted questions and websites, with a third blinded reviewer resolving discrepancies.

**Table 1:** Adapted Rothwell classification An adapted version of the Rothwell classification system, similar to the one used by Suresh et al. [7,16], was used for question and website designations.

Adapted Rothwell classification criteria	
Question categorization	
Fact	
Cost	Financial aspects of the procedure, including out-of-pocket expenses and payment plans.
Timeline of recovery	Duration and stages of recovery post-surgery.
Restrictions	Limitations on activities or lifestyle during the recovery period or indefinitely.
Risk of complications	Potential adverse effects or complications that may arise during or after the procedure.
Technical details	The surgical procedure itself, including methods, tools, and anesthesia.
Insurance coverage	Whether the procedure is covered by insurance and the extent of coverage.
Underlying condition	Medical conditions that necessitate the surgery and their management.
Policy	
Indications	Criteria and medical indications for undergoing the procedure.
Follow-up care	Necessary post-operative care, including follow-up appointments and care instructions.
Preoperative preparation	Steps and preparations required before undergoing the procedure.
Value	
Alternative treatments	Other treatment options available instead of the procedure.
Cosmetic concerns	Aesthetic outcomes and appearance post-surgery.
Pain	Pain management during and after the procedure.
Website categorization	
Government	Ending in .gov or maintained by a national or state government.
Commercial	Maintained by commercial organizations, often providing health information and associated with companies or products.
Academic	Affiliated with universities, academic medical centers, or academic societies.
Medical practice	Local hospitals or medical practices without broader academic affiliation.
Single surgeon personal	Created and maintained by individual surgeons, often highlighting personal practices and patient information.

Journal of the American Medical Association quality assessment

The Journal of the American Medical Association (JAMA) Benchmark Criteria, a set of standards established to ensure quality online information, was employed to assess the quality of websites associated with the PAA questions, and a panel of reviewers assigned JAMA scores [17]. A maximum score of 4 would be assigned if the website met all four benchmark criteria: authorship, attribution, currency, and disclosure. Any score below 3 would indicate that the source may be of questionable quality [17].

Integration of framework for LLM use

**Figure 1:**
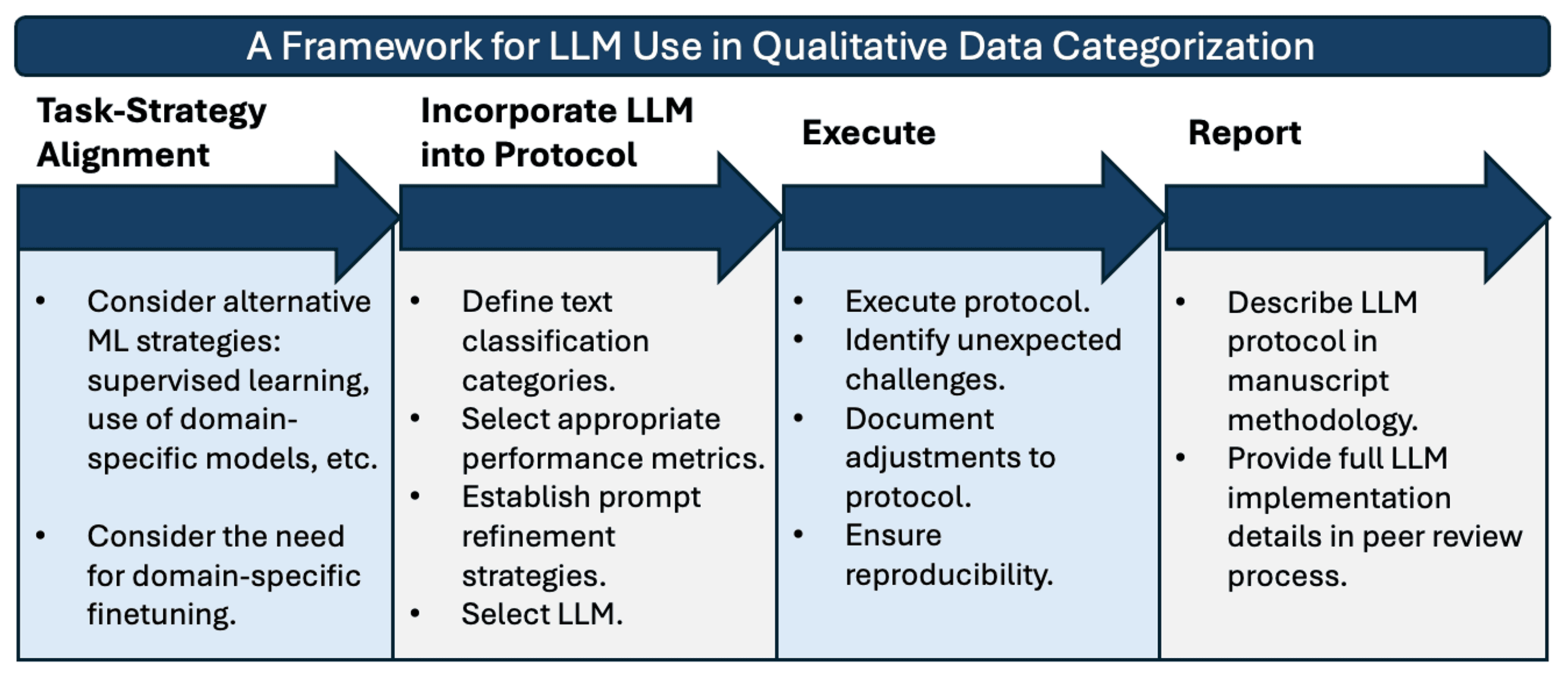
A framework for LLM use in qualitative data categorization A novel extension of the model framework established by Hasan et al. [15]. LLM, large language model

We extended the framework by Hasan et al. to the categorization of qualitative data through LLMs (Figure 1) [15]. The framework was designed to ensure alignment of the research task and strategy, maintain methodological rigor, and promote transparency in reporting.

The proposed internet search analytics use case was determined to be appropriate given the unlabeled and general nature of the input data and the alignment of the classification task with the model's training objectives, both obviating the need for domain-specific fine-tuning. The previously determined MMS question subcategories were used for classification targets. GPT-4 was chosen for its performance and efficiency in the text classification task. Prompts were iteratively refined to optimize output quality and minimize hallucinations. Metrics, including percent agreement and Cohen’s kappa, were established, and the protocol was implemented with results prepared for reporting.

Classification models

Three algorithms for question classification were used as a comparison to LLM integration: KNN cluster models, DTCs, and LR models. The training dataset was derived from PAA-extracted question and subcategory label pairs. For feature extraction, question text was embedded in a semantically meaningful numerical space by three high-performing, pre-trained embedding models with competitive performance on the Massive Text Embedding Benchmark. Selected embedding models include gte-base-en-v1.5, all-MiniLM-L6-v2, and Google's nnlm-en-dim128 [18-20] (Embedding model: Google. NNLM: Neural Network Language Model. Kaggle; October 6, 2020). LLM classification was done via zero-shot, one-shot, and multi-shot learning approaches, with randomly and researcher-selected question label pairs. Percent agreement in question categorization was determined for all algorithms, with Cohen’s Kappa selectively evaluated for LLM performance.

Statistical analyses

Descriptive statistics and Pearson’s chi-square test for independence were used to analyze nominal data, specifically analyzing the relationship between search terms and question types and between search terms and website type. Four questions (cost, preoperative care, insurance coverage, and pain) and one website type (single surgeon personal) were omitted from Pearson’s chi-square test to ensure constraints and assumptions regarding adequate sample size were met. Post hoc adjusted (standardized) residual analysis was performed to identify points of significance. Significance thresholds for interpretation of the residuals include the following: residuals greater than ±1.96 correspond to a p-value of 0.05, residuals greater than ±2.58 correspond to a p-value of 0.01, and residuals greater than ±3.29 correspond to a p-value of 0.001.

To compare the performance of the different models, a Z-score test for two population proportions with a two-tailed hypothesis was performed. Student’s t-tests and ANOVA were used to compare JAMA Benchmark scores by website type and search term. Statistical significance was set to p-values <0.05. Statistical analyses were performed using Microsoft Excel Version 16.80 (Microsoft Corp., Redmond, WA). ANOVAs were conducted using the Analysis ToolPak with Microsoft Excel.

## Results

JAMA quality assessment and trends

**Figure 2:**
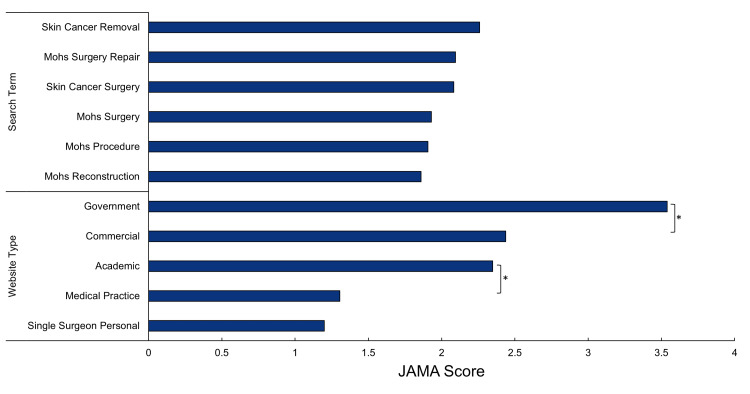
JAMA scores by website type and search term query *Indicates p-value < 0.001 as determined by Student's t-test with two-sample equal variance. JAMA, Journal of the American Medical Association

**Figure 3:**
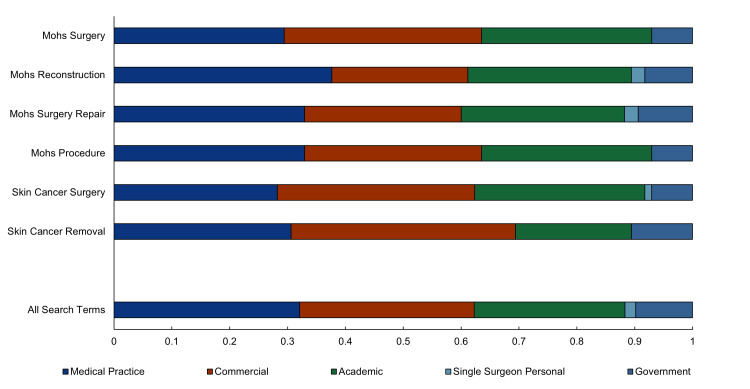
Proportions of website types returned by each search term query Statistical analysis revealed no significant association in the distribution of websites for each search term (p > 0.05).

The average JAMA score earned across all 265 unique websites returned by the six search terms was 2.02 ± 1.14. Websites most often failed to garner points in the authorship and attribution standards. The ANOVA test conducted on JAMA scores by search terms suggested no statistical difference among results for each search term (p > 0.10) (Figure 2). Government sites had the highest JAMA scores (3.53 ± 0.99) but represented only 10% of the websites. The most frequently referenced website category, medical practices (32.1%), displayed significantly lower JAMA scores (p < 0.01) than academic, government, or commercial websites. Proportions of website types are displayed in Figure 3. Both Pearson’s chi-square test for independence and post hoc adjusted residuals revealed no significant association in the distribution of websites for each search term.

Prevalence of PAA question types

**Figure 4:**
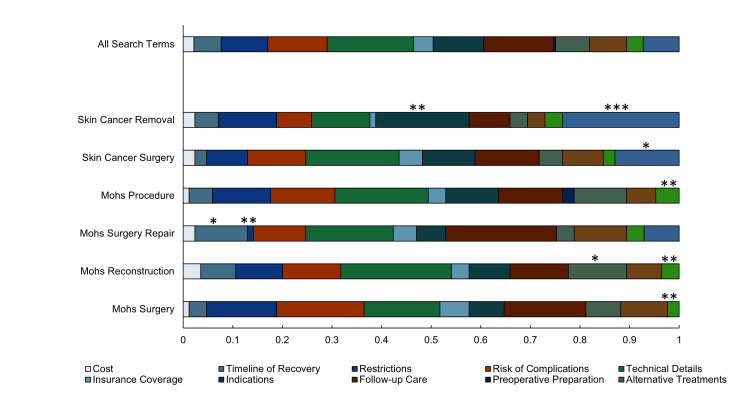
Proportions of question types returned by each search term query Significance as determined by adjusted (standardized) residual analysis is indicated: ***p < 0.001, **p < 0.01, and *p < 0.05.

The application of modified Rothwell categorizations yielded the following percentages of questions for fact, policy, and value, respectively: 58.7%, 24.8%, and 16.5%. Of the 303 unique questions from the query, the highest proportion of questions belonged to technical details (19.8%), follow-up care (12.5%), indications (11.6%), risk of complications (10.9%), and underlying condition (10.2%) (Figure 4). Of the questions that regarded technical details, they frequently pertained to the depth of the surgical incision, procedure length, and anesthesia administration. Question categories with the least representation included the following: preoperative preparations (0.7%), cost (1.7%), insurance coverage (2.3%), and pain (3.3%).

The most commonly asked question was “What are the disadvantages of Mohs surgery?” The next five most commonly asked questions were the following: “Does Mohs surgery leave a hole?”, “How deep do they cut for basal cell carcinoma?”, “What is the life expectancy of a person with basal cell carcinoma?”, “What not to do after Mohs surgery?”, and “Will I be disfigured after Mohs surgery?”.

Pearson’s chi-square test for independence revealed a significant association between question type and search term (p < 0.001), indicating that the search term had a significant effect on the distribution of PAA question categories that were returned by the query. Furthermore, post hoc analysis showed where these significant differences in questions lie (Figure 4).

Comparison of classification models

**Figure 5:**
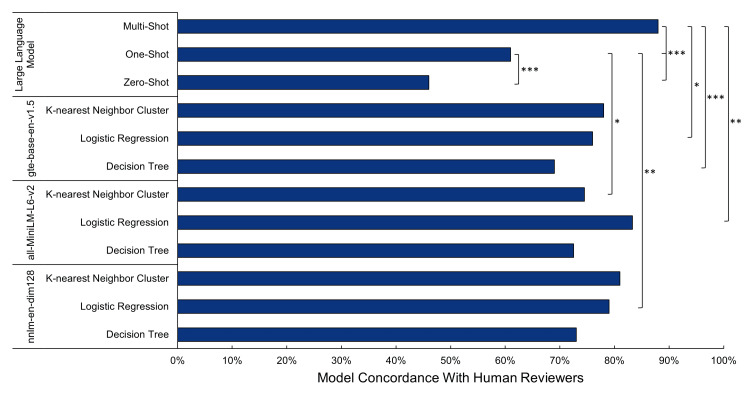
Performance of selected classification models Statistical significance as determined by the Z-score test for two population proportions is indicated: ***p < 0.001, **p < 0.01, and *p < 0.05.

The applied ML algorithms of this study were selected due to their availability via well-curated and documented software packages [21-22]. The classification models averaged 71-78% classification accuracy between the different provided feature embeddings (Figure 5). The performed Z-score test for two population proportions indicated no significant statistical difference across any of the model-embedding pairings (p > 0.05). LLM-based classification showed the highest overall accuracy and increased consistently with expanded prompts, achieving 46% (zero-shot), 66% (one-shot), and 88% (multi-shot) labeling accuracy. Multi-shot performance was found to be statistically greater than either the one-shot or the zero-shot (p < 0.001) as well as all embedding-feature extraction models (p < 0.05), except for gte-base-en-v1.5 KNN, all-MiniLM-L6-v2 LR, nnlm-en-dim128 KNN, and nnlm-en-dim128 LR. When evaluated using Cohen’s kappa to measure inter-rater reliability between the LLM and the human panel of reviewers, kappa values of 0.40 (zero-shot), 0.57 (one-shot), and 0.86 (many-shot) were observed.

## Discussion

In the context of healthcare, the internet continues to grow in its role of providing information [5]. Google’s PAA algorithm provides an efficient way for the analysis of online users’ questions. Surveying patients directly in healthcare settings may seem logical but can introduce bias due to discomfort asking questions in front of providers [23]. Google’s PAA feature allows for investigation of concerns that patients may be unlikely to share.

Most questions searched related to MMS concerned the technical details of the procedure, namely procedure length, anesthesia, and incision specifics. Thus, surgeons should spend adequate time explaining those technical details to patients prior to surgery to ensure that patients are comfortable proceeding. Queries pertaining to the financial aspect of surgery, such as cost and insurance coverage, were much less common, indicating that pecuniary aspects concerned patients less.

Our analysis showed significant differences in question type distribution by each of the search terms. Specifically, the significant correlation between “skin cancer removal” and question types “indications” and “underlying conditions” implies that individuals using this term may be earlier in the decision-making process or more concerned about the medical necessity of the surgery and its connection to their general health. This insight could guide clinicians to address these informational needs more directly for patients interested in or considering MMS as a treatment for skin cancer. Search terms did not result in significant differences in website-type distribution.

We also identified the corresponding sources associated with the PAA questions and their quality. The average JAMA score of all the websites, 2.02 ± 1.14, indicated that the information patients find online regarding MMS may be of questionable quality, lacking in either authorship, attribution, currency, or disclosure. JAMA scores did not vary significantly from the websites returned from one search term to the next. This indicates that a patient with lower health literacy searching “skin cancer removal” is no more likely to encounter poor quality sources than a more health literate patient who uses the “Mohs surgery” search term.

The greatest number of websites associated with PAA questions were medical practices. Unfortunately, medical practices were associated with lower JAMA quality benchmark scores. Conversely, the government sites were associated with much higher JAMA scores yet represented a much smaller proportion of websites associated with PAA questions.

The integration of ML algorithms demonstrated promising potential in extracting clinically relevant insights from patient-facing data. The ML algorithms were consistently outperformed by the LLM multi-shot learning approach, with improvements in test accuracy ranging from 9% to 17% (Figure 5). This difference in performance may be attributed to the LLM's pretraining on a vast collection of text, improving its ability to capture semantic nuances. Notably, the multi-shot learning approach resulted in a statistically significant increase in percent agreement with human reviewers as compared to either zero-shot or one-shot learning approach. This highlights the relevance of appropriate prompt engineering and refinement. Inadequate prompt preparation, testing, and adjustment will result in decreased accuracy of qualitative applications. The ML algorithms outperformed the LLM when one-shot and zero-shot learning were employed. The LLMs appear to leverage additional context provided by a multi-shot prompt, resulting in higher accuracy when categorizing qualitative data, even with potential label overlap. This approach enhanced their ability to resolve ambiguities and improve agreement rates and inter-rater reliability.

While the application of ML algorithms requires manual feature extraction - here, the use of semantic embedding models - to provide appropriate mathematical input, the LLM integrates this step directly into its architecture, allowing for greater optimization of individual components, more efficient training, and higher downstream performance. These reductions in researcher-facing complexity further reinforce the usefulness of LLMs in research protocols. Especially in interdisciplinary or highly specialized projects, the increased accessibility of LLM models is a noteworthy advantage.

LLMs are often evaluated with quantitative error metrics to communicate performance on specific downstream tasks. However, it should be noted that in the context of qualitative data analysis, it may be problematic to assign concrete ground truth labels due to inherent overlap between categories, such as between “cost” and “insurance coverage.” The rationale justifying the use of a human panel for a consensus opinion therefore complicates the task of LLM classification and may cause poor representation of LLM performance. Notably, the LLM performance of 88% accuracy per panel-assigned labels exceeds average individual reviewer agreement with the panel (80%), suggesting that the multi-shot LLM is more consistent with the panel than either reviewer would be individually.

Furthermore, LLMs are not subject to data collection fatigue. Thus, in properly powered studies with a high volume of data to be collected and reviewed, an appropriately trained LLM would vastly outperform a standard human reviewer in consistency metrics.

Per these considerations, Cohen’s kappa may better represent LLM performance in qualitative data applications, and future directions should prioritize such metrics in LLM evaluation. These and other measures may adequately account for subjectivity and bias in human panels while allowing for greater generalizability of results. LLM many-shot performance exhibited a kappa value of 0.86, which can be interpreted to indicate near-perfect agreement [24]. These findings, jointly with the demonstrated 88% agreement, suggest that LLMs can reliably replicate human consensus opinions in limited qualitative analysis tasks, reinforcing their potential as an essential tool for scaling up data analysis processes without compromising precision. Prioritization of Cohen’s kappa, as the evaluation of inter-rater agreement, naturally suggests the future use of LLM outputs in conjunction with human reviewers as a method to mitigate the limitations of both. However, it is important to acknowledge that while LLMs may reliably replicate human judgment, they are dependent on accurate training sets. Biased, incomplete, or inaccurate training sets will propagate errors into results.

While Google’s PAA function does allow for the analysis of common queries, it does not provide information on who submitted the queries. This study relies upon the assumption that those asking questions regarding MMS are patients, although it is likely that a portion of those using the search terms analyzed in this study were not patients and may have been healthcare workers, students, or kin of the patient. Additionally, this study may suffer from sampling bias by failing to properly account for the questions of patients of low socioeconomic status (SES). They are less likely to use internet health resources as compared to their high SES counterparts, and the results of this study may exclude some of the questions and concerns that low SES patients may have [25]. Any SES patient queries directed through non-internet media may contribute to the underrepresentation of patient concerns in Google PAA data.

It is worth noting that while JAMA criteria were designed as a standard of quality for online material, its creators have conceded that a high JAMA score does not ensure that the information the source provides is reliable [17]. Furthermore, Silberg et al. claim that quality information can come from other sources, such as online learning platforms and crowdsourced knowledge bases, that typically would garner lower JAMA scores [17]. Thus, low JAMA scores do not necessarily mean that the information is of poor quality. Despite its drawbacks, the JAMA benchmark does provide a foundation by which different sources can be compared on the basis of authorship, attribution, disclosure, and currency.

## Conclusions

MMS patients are interested in understanding the technical details of their condition and the procedure. Healthcare professionals should take action to address these concerns to ensure greater compliance with treatment protocol and mitigate the risk of iatrogenic trauma. Additionally, patients are more likely to find quality information on government sites and are more prone to finding material of lower quality on websites associated with medical practices or single surgeons. We propose that in qualitative research applications involving a panel of reviewers, high-performing LLMs offer a suitable component of a review board and a complement to human expertise, improving the efficiency and robustness of these analytical approaches.

## References

[1] Johnstone C, Joiner KA, Pierce J, Krouse RS (2018). Mohs micrographic surgery volume and payment patterns among dermatologists in the Medicare population, 2013. Am J Clin Oncol.

[2] Muzic JG, Schmitt AR, Wright AC (2017). Incidence and trends of basal cell carcinoma and cutaneous squamous cell carcinoma: a population-based study in Olmsted County, Minnesota, 2000 to 2010. Mayo Clin Proc.

[3] Rowe DE, Carroll RJ, Day CL Jr (1989). Long-term recurrence rates in previously untreated (primary) basal cell carcinoma: implications for patient follow-up. J Dermatol Surg Oncol.

[4] Murray C, Sivajohanathan D, Hanna TP (2019). Patient indications for Mohs micrographic surgery: a systematic review. J Cutan Med Surg.

[5] van Egmond S, Wakkee M, Hoogenraad M, Korfage IJ, Mureau MA, Lugtenberg M (2022). Complex skin cancer treatment requiring reconstructive plastic surgery: an interview study on the experiences and needs of patients. Arch Dermatol Res.

[6] Devlin J, Chang MW, Lee K, Toutanova K (2019). BERT: pre-training of deep bidirectional transformers for language understanding [PREPRINT]. Assoc. Comput. Linguist.

[7] Suresh N, Fritz C, De Ravin E, Rajasekaran K (2024). Modern internet search analytics and thyroidectomy: what are patients asking?. World J Otorhinolaryngol Head Neck Surg.

[8] Breneman A, Gordon ER, Trager MH, Ensslin CJ, Fisher J, Humphreys TR, Samie FH (2024). Evaluation of large language model responses to Mohs surgery preoperative questions. Arch Dermatol Res.

[9] Hernandez LE, Mohsin N, Does AV, Martin M, Saaraswat M, Dreyfuss I, Nouri K (2023). Assessing online patient education materials in dermatology: a call to action. Arch Dermatol Res.

[10] Onyemachi J, Pinto-Cuberos J, Miller D, Wagner RF, Winsett F (2024). 3D models for mohs micrographic surgery: a review on its use in patient education. Arch Dermatol Res.

[11] Patel P, Malik K, Khachemoune A (2021). Patient education in Mohs surgery: a review and critical evaluation of techniques. Arch Dermatol Res.

[12] Scheinkman R, Kraft G, Kasheri E, Latta S, Papavasilopoulos R, Nouri K (2024). An analysis of the quality of online mohs micrographic surgery postoperative patient instructions. Arch Dermatol Res.

[13] Archibald LK, Ascha MS, Bordeaux JS (2021). Why it's hard to see a dermatologist for a skin exam: the patient perspective. Arch Dermatol Res.

[14] Kamath P, Kursewicz C, Ingrasci G, Jacobs R, Agarwal N, Nouri K (2019). Analysis of patient perceptions of Mohs surgery on social media platforms. Arch Dermatol Res.

[15] Hasan B, Saadi S, Rajjoub NS (2024). Integrating large language models in systematic reviews: a framework and case study using ROBINS-I for risk of bias assessment. BMJ Evid Based Med.

[16] Rothwell JD (2013). In Mixed Company: Communication in Small Groups.

[17] Silberg WM, Lundberg GD, Musacchio RA (1997). Assessing, controlling, and assuring the quality of medical information on the Internet: Caveant lector et viewor--Let the reader and viewer beware. JAMA.

[18] Zhang X, Zhang Y, Long D (2024). mGTE: Generalized Long-Context Text Representation and Reranking Models for Multilingual Text Retrieval [PREPRINT]. arXiv.

[19] Wang W, Wei F, Dong L, Bao H, Yang N, Zhou M (2020). MiniLM: deep self-attention distillation for task-agnostic compression of pre-trained transformers [PREPRINT]. arXiv.

[20] Li Z, Zhang X, Zhang Y, Long D, Xie P, Zhang M (2023). Towards general text embeddings with multi-stage contrastive learning [PREPRINT]. arXiv.

[21] Abadi M, Agarwal A, Barham P (2015). TensorFlow: large-scale machine learning on heterogeneous systems [PREPRINT]. arXiv.

[22] Pedregosa F, Varoquaux G, Gramfort A (2011). Scikit‐learn: machine learning in python. J Mach Learn Res.

[23] Katz MG, Jacobson TA, Veledar E, Kripalani S (2007). Patient literacy and question-asking behavior during the medical encounter: a mixed-methods analysis. J Gen Intern Med.

[24] McHugh ML (2012). Interrater reliability: the kappa statistic. Biochem Med (Zagreb).

[25] Kontos E, Blake KD, Chou WY, Prestin A (2014). Predictors of eHealth usage: insights on the digital divide from the Health Information National Trends Survey 2012. J Med Internet Res.

